# Randomized controlled trial to treat migraine with acupuncture: design and protocol

**DOI:** 10.1186/1745-6215-9-57

**Published:** 2008-10-20

**Authors:** Ying Li, Fanrong Liang, Shuguang Yu, Xuguang Liu, Yong Tang, Xuguang Yang, Xiaoping Tian, Jie Yan, Guojie Sun, Xiaorong Chang, Hui Zheng, Hongxing Zhang, Tingting Ma

**Affiliations:** 1 Chengdu University of Traditional Chinese Medicine, Chengdu, Sichuan, PR China; 2 Hunan University of Traditional Chinese Medicine, Changsha, Hunan, PR China; 3 Hubei College of Traditional Chinese Medicine, Wuhan, Hubei, PR China; 4 No.1 People's Hospital of Wuhan City, Wuhan, Hubei, PR China

## Abstract

**Background and motivation:**

The effectiveness of using acupuncture to treat migraine is rarely and even suspectedly reported in the literature. In this article, we report the design and the protocol of a randomized controlled large-scale trial to treat migraine using acupuncture, aiming at testifying it is effective to use acupuncture to treat migraine. We demonstrate also that the effectiveness of the treatment may vary due to using acupoints of different meridians or different acupoints of one meridian.

**Methods and design:**

A multi-center randomized controlled trial is currently undergoing, with three acupoints treatment groups and one non-acupoints control group. The acupuncture treatment consists of 20 sessions per patient with a observation period of 20 weeks. In total, 480 patients with Migraine are registered in this study within 8 hospitals in China from March 2008 to June 2009. These patients are randomly assigned to receive one of the following four acupoints treatment groups, i.e. 1) specific acupoints of Shaoyang meridians (120 patients), 2) non-specific acupoints of Shaoyang meridians (120 patients), 3) acupoints of other meridians (120 patients); or 4) non-acupoints control group (120 patients). The main outcome measurement in this trial is the effect comparison achieved among these four groups in terms of number of days with migraine and intensity of migraine during and after the baseline phase, i.e. the first 4 weeks before randomization and 4, 8 and 16 weeks after randomization. The intensity of headache including Headache intensity grade (0–3) and visual analogue scale (VAS) score will also be used in this study. In addition, the differences of Migraine-Specific Quality-of-Life Questionnaire (MSQ) and Transcranial Doppler Sonography (TCD) before and after randomization are also used as the secondary outcome measurement.

**Discussion:**

The result of this trial (which will be available in 2009) will demonstrate the efficacy of using acupuncture to treat migraine, and verify whether the specific effect of acupoints exists and whether this specific effect of acupoints is related to meridian and a collection of meridian Qi.

**Trials registration:**

Clinical Trials.gov NCT00599586

## Background

Migraine is a common disease. Population-based studies suggest that 6% to 7% of men and 15% to 18% of women experience migraine headache [1,2]. Acupuncture is gradually accepted as a complementary or alternative medicine for preventing migraine attack and relieving pain in countries beyond China, especially in the West. Recently, a randomized trial showed that acupuncture leads to persisting, clinically relevant benefits for patients with chronic headache, particularly migraine [3], which is consistent with many results reported in the previous literature on acupuncture for headache [4-6].

However, in recent years several randomized controlled trials argued that acupuncture was no more effective than sham acupuncture or minimal acupuncture in reducing migraine headache and tension-types of headache, although it was more effective than no treatment [7,8]. Convinced evidence for the efficacy of treating migraine with acupuncture is therefore inadequate because of these inconsistent results. In this multi-center randomized controlled large-scale trial, we firstly aim at investigating whether acupuncture is effective to treat migraine through a comparison with non-acupoints control. Secondly we investigate whether its efficacy is dissimilar due to using acupoints of different meridians or different acupoints of one meridian. The work reported in this article is financed by the National Basic Research Program (***973 Program***) in China, and is registered with an identifier (NCT00599586) by Clinical Trials.gov in the USA.

## Methods and design

### Design

**Figure 1:**
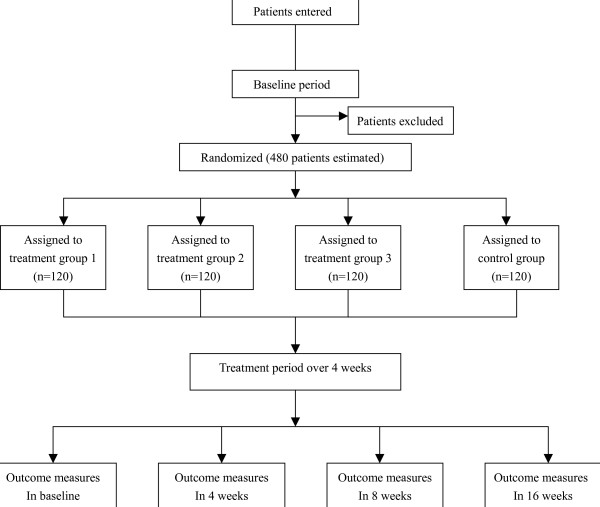
Trial flow chart.

**Figure 2:**
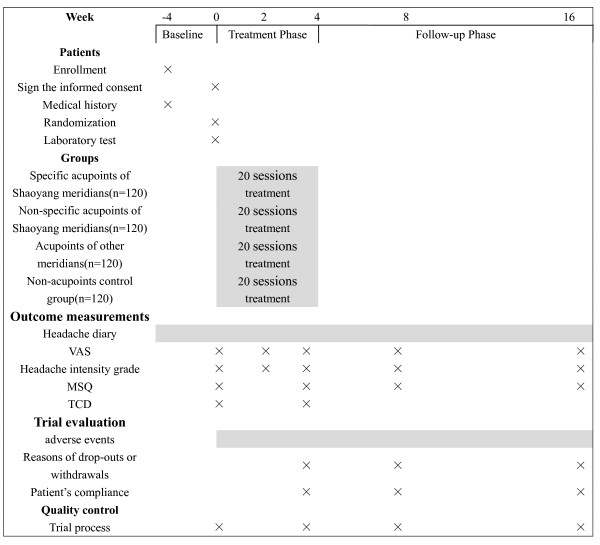
**Trial processes chart**. Note: VAS = visual analogue scale (0–10); MSQ = Migraine-Specific Quality-of-Life Questionnaire; Headache intensity grade: 0 = no headache; 1 = mild headache; 2 = moderate headache; 3 = severe headache; TCD = Transcranial Doppler Sonography.

This study is a multi-center randomized controlled trial comparing three acupoints treatment groups with one non-acupoint control group (fig. 1, fig 2). The design of this study followed the guidelines of the HIS Committee on Clinical Trails in Migraine [9]. The trial is completed in the following eight hospitals: First affiliated Hospital of Chengdu University of Traditional Chinese Medicine (TCM); People's Hospital of Sichuan province; No.4 People's Hospital of Sichuan province; Affiliated Hospital of Ningxia Medicine University; First affiliated Hospital of Hunan University of TCM; People's Hospital of Hunan province; No.1 People's Hospital of Wuhan City; TCM Hospital of Wuhan City.

The total observation period within this study is 20 weeks for each patient. All patients should have recorded headache diaries for 4 weeks before randomization (baseline phase). After randomization the patients will receive 20 sessions of 30 minutes' duration over a period of 4 weeks. All patients are asked to document their headache diaries, and the outcome measurement is completed both in the baseline and 4, 8 and 16 weeks after randomization.

The patients in the acupoints treatment groups and non-acupoint control group are blinded as to which treatment method they received. The patients will be nevertheless informed that they will receive either one of acupoints treatment groups or non-acupoint control group, which has been associated with positive outcome in clinical studies.

The central randomization is used in this trial, which is performed by the National Clinical Trial Center of Chinese Medicine, Chengdu, Good Clinical Practice (GCP) Center in China. The patients meeting the inclusion criteria will be randomly assigned to one of acupoints treatment groups or non-acupoint control group by the GCP Center through telephone, mobile phone, mobile message, or website, and a random number and group assignment will be immediately confirmed through email. This procedure assures that randomization will not be influenced, neither by the acupuncturists nor the patients.

This trial is performed according to the principles of the Declaration of Helsinki (Version Edinburgh 2000). And the trial protocol has been approved by local institutional review board and ethics committee. The written informed consent will be requested to sign for all of participants before registration. All patients will be given enough time to decide whether they would sign the informed consent, or they will be given treatment options other than acupuncture if they are not willing to participate.

### Patients

A power analysis is calculated before conducting this trial, which *α *= 0.05 and power = 90%. According to the previous literature [7], for the main outcome the number of days with migraine, an improvement of 2.4 days by sham acupuncture is reported. In this study, we anticipated an improvement of 4.0 days by acupuncture, so a difference of 1.6 days is considered minimally of clinical significance. Therefore, the sample size in this trial can be estimated initially according to the following formula [10]. Each group needs at least 120 patients (assuming a 15% dropout rate), 480 patients in four groups totally will be enrolled in the study.

$$n=\frac{\psi^{2}(\sum_{j=1}^{k}s_{j}^{2}/k)}{\sum_{j=1}^{k}{\left({\bar{x}}_{j}-\bar{\bar{x}}\right)}^{2}/(k-1)}$$

### Inclusion criteria

According to the criteria of the International Classification of Headache Society [11], patients who meet the diagnosis of migraine with or without aura will include in this study. The criteria are: 1)suffering from acute attacks of migraines for at least 1 year, with two or more migraine attacks per month during the last three months and during the baseline period; 2) between 18 and 65 years old with the onset age of migraine less than 50; 3)with baseline headache diary completed; 4) without using prophylactic drugs in the previous 1 month, 5) willing to finish 20 treatments in the following 4 weeks (acupuncture treatment period); and 6) with written consent form signed by themselves.

### Exclusion criteria

Patients with any of the following conditions will be excluded: headache caused by organic disorders, such as subarachnoid hemorrhage, cerebral hemorrhage, cerebral embolism, cerebral thrombosis, vascular malformation, arteritis, hypertension, arteriosclerosis, etc.; psychosis; pregnant women or women in lactation; patients with allergic constitution or easily getting infected or bleeding; or patients having taken drugs for preventing migraine in the last four weeks.

### Interventions

It took at least twelve months to develop the treatment strategies for this study. Interventions were according to records in ancient books and research results of modern papers about treating migraine with acupuncture in China or in the West, and were formed in consensus with Chinese acupuncturists and acupuncture experts, who come from both the study team and non-study team in all regions where the study is being conducted. In the first part of the clinical research of our ***973 project ***(accomplished in 2007), we confirmed the location of non-acupoints through compared one acupoints group with two non-acupoints control groups. The final strategies for this study are therefore defined as three acupoints treatment groups and one non-acupoints control group.

Based on the theory of TCM and acupuncture, migraine pertains to Shaoyang headache, and it has a close relationship with Shaoyang meridians, while it dose not relate to other meridians. Therefore, the effect of Shaoyang meridian is different from that of other meridians in acupuncture treatment of migraine. Additionally, in the same meridian, the effect of specific acupoints is different from the non-specific acupoints. Therefore, in our trial the three acupoints treatment groups are defined as 1) treatment group 1 (specific acupoints of Shaoyang meridians); 2) treatment group 2 (non-specific acupoints of Shaoyang meridians), and 3) treatment group 3 (acupoints of other meridians), and one non-acupoints control group.

#### Treatment group 1

Waiguan (TE5), Yanglingquan (GB34), Qiuxu (GB40), Fengchi (GB20) are punctured by filiform needles unilaterally. Waiguan (TE5) is punctured perpendicularly 0.5–1 cun. Yanglingquan (GB34) is punctured perpendicularly 1–1.5 cun. Qiuxu (GB40) is punctured perpendicularly 0.5–0.8 cun. Fengchi (GB20) is punctured obliquely 0.8–1.2 cun, the tip of needle towards the tip of the nose.

#### Treatment group 2

Luxi (TE19), Sanyangluo (TE8), Xiyangguan (GB33), Diwuhui (GB42) are punctured by filiform needles unilaterally. Luxi (TE19) is punctured transversely 0.3–0.5 cun. Sanyangluo (TE8) is punctured perpendicularly 0.5–1 cun. Xiyangguan (GB33) is punctured perpendicularly 1–1.5 cun. Diwuhui (GB42) is punctured perpendicularly 0.5–0.8 cun.

#### Treatment group 3

Touwei (ST8), Pianli (LI6), Zusanli (ST36), Chongyang (ST42) are punctured by filiform needles unilaterally. Touwei (ST8) is punctured transversely 0.5–1.0 cun. Pianli (LI6) is punctured perpendicularly 0.3–0.5 cun. Zusanli (ST36) is punctured perpendicularly 1–2 cun. Chongyang (ST42) is punctured perpendicularly 0.3–0.5 cun, avoid needling the artery.

#### Control group

In the medial arm on the anterior border of the insertion of the deltoid muscle at the junction of deltoid and biceps muscles [12], is punctured perpendicularly 0.5–1 cun. The edge of tibia 1 to 2 cm lateral to the Zusanli (ST36) horizontally [13], is punctured perpendicularly 0.5–1 cun. Half between the tip of the elbow and the axilla [14], is punctured perpendicularly 0.5–1 cun. Ulnar side, half between epicondylus medialis of the humerus and ulnar side of the wrist [14], is punctured perpendicularly 0.5–1 cun. All non-acupoints are punctured by filiform needles unilaterally.

In each session all acupoints and non-acupoints are punctured by filiform needles unilaterally, and left and right side are alternatively used. After 4 acupoints or non-acupoints being needled, 4 auxiliary needles will be punctured at 2 mm lateral to every acupoint or non-acupoint and punctured to a depth of 2 mm without manual stimulation. Transcutaneous electric acupoint stimulation (HANS: Han's acupoint nerve stimulator, HANS-200, made in Nanjing, China) is used for electro-acupuncture stimulation at every acupoint or non-acupoint after needle insertion. Each acupuncture needle of acupoints or non-acupoints and its auxiliary needle are connected with the electricity by HANS for 30 minutes. The stimulation frequency is 2/100 Hz. The stimulation intensity varied from 0.1 mA to 1.0 mA until the patients feel comfortable.

The filiform needles are used in this trial, which are sterile acupuncture needles for single use; namely Hwato Needles, made in Suzhou, China, 25–40 mm in length and 0.25 mm in diameter. The needles with 13 mm in length and 0.18 mm in diameter especially are used as auxiliary needle without manipulating the needles. The acupoints of the treatment groups are asked to achieve DeQi sensation by lifting and thrusting combined with twirling and rotating the needles, but the non-acupoints of control group are not asked to achieve DeQi sensation by the same manipulating the needles. Both the treatment groups and control group are asked to retain the needles for 30 minutes, and close the acupoint holes with clean cotton balls to avoid bleeding when withdrawing the needle.

All patients will receive 20 treatments totally over a period of 4 weeks, once a day, five times continuously and two days interval in a week.

### Outcome measurement

The main outcome measures in the trial are the differences between the number of days with migraine and intensity of headache in the headache diaries during 4 weeks before randomization and 4, 8, and 16 weeks after randomization. The intensity of headache includes the Headache intensity grade (0–3) and visual analogue scale (VAS) score (0–10). The secondary Outcome Measures are the differences in frequency of migraine attacks (episodes of migraine headaches separated by pain free intervals of at least 48 hours), average duration of a migraine attack, rate of rescue medication used, number of patients with adverse side effects, Migraine-Specific Quality-of-Life Questionnaire (MSQ) before randomization and 4, 8, and 16 weeks after randomization, and the differences in Transcranial Doppler Sonography (TCD) before randomization and 4 weeks after randomization.

All patients fill in headache diaries during 4 weeks before randomization (baseline phase) and 4, 8, and 16 weeks after randomization. In the diaries they document the time of attack and stopping of headache, the intensity, the frequency, the location and the cause of headache, associated symptoms in each migraine attack. The patients document whether or not they take the medicine during treatment periods, and if patients take medicine they have to document the name, dosage of the medicine, the time of taking medicine, the time of relieving pain and the side effect of the medicine. In addition, all patients will accept routine test of blood, urine and stool, and electrocardiogram (ECG), liver function (ALT, AST), kidney function (BUN, Scr) before randomization, in order to exclude these patients who have serious illness in heart, liver and kidney or other severe disease.

Any adverse events, and how they are addressed, are recorded during the four treatment weeks and twelve follow-up weeks. These adverse events include bleeding, hematoma, fainting, serious pain, and local infection. If patients suffer any serious adverse events, all details will be documented.

All acupuncturists are required to attend special training classes in order to understand the details of this trial. The special training classes have theoretical lessons and practical lessons. All people have to understand the purpose and content of the trial, treatment strategies and quality control. For instance, they are trained to know how to use the central randomization method, how to fill in the CRF (case report form) and electro-CRF, how to locate the points and manipulate the needles, etc. The acupuncturists must take part in all of the training classes as well and have to pass the special training test before they are qualified to perform this trial. Additionally, in order to keep quality control, a special quality examination form is made for checking the whole processes of the trial and documenting the details of the processes. This work is performed by the special trained checkers once a month in every hospital in the centers.

Drop-outs or withdrawals and their reasons, and patient's compliance have to be documented during the four treatment weeks and twelve follow-up weeks. All patients are recorded until the end of the trial.

### Statistical analysis

Analysis of all data in this trial will be performed by the National Clinical Trial Center of Chinese Medicine (Chengdu GCP Center) in China, which is performed in a blinded manner because analysis of data is done by a special analysis-researcher. The data will be analyzed with SPSS13.0 and SAS9.0 statistical software packages.

The intention-to-treat (ITT) population is defined as the patients who are randomized. The data analysis of baseline characteristics is based on the intention-to-treat (ITT) population. And the data analysis of the primary and secondary outcomes and other outcomes is mainly based on the intention-to-treat (ITT) population. In addition the per-protocol (PP) population is analyzed. The result of the ITT analysis will be compared with that of the PP analysis to check whether the results are consistent.

The first step of analysis is to make comparisons among baseline characteristics in different groups. The second step is to check whether acupoints treatment groups were more efficacious in treating migraine than non-acupoints control group. If acupoints treatment is more effective than non-acupoints treatment in treating migraine, the third step is to make comparisons among three acupoints treatment groups in effectiveness of treating migraine.

For normal distribution data (normality, homogeneity of variances) the repeated measures analysis will be used in the different time-points assessment. The Kruskal-Wallis test will be employed in the analysis of skewed distribution data. Moreover, analysis of variance (ANOVA) and used for numerical variables, Chi-square test is used for categorical variables. Two-sided test is applied for all available data, and a P value < 0.05 is considered statistically significant.

## Discussions

The National Basic Research Program (***973 Program***) is the most important basic research program based on clinical practice in China. Our project is the largest clinical trial involved in the specific physiological effects of acupoints, financially supported by the ***973 Program***. Because of a controversial issue reported in other studies about the specific effect of acupoints, our project aims to clarify this issue by means of a multi-center randomized controlled trial.

The study reported in this article is the second part of our ***973 project***. It involves in comparing the efficacy of acupoints treatment with non-acupoints treatment and the efficacy of different acupoints treatment, after efficacy of acupuncture treating migraine is convinced from the first part of our project.

According to the theory of TCM and acupuncture, there is no doubt that the differences between acupoints and non-acupoints in specific physiological effects do exist. Acupuncture has also been recognized as a method for treating many diseases, not only widely in China due to its long history in clinical practice, but also gradually in many western countries due to its increasing popularity. However, in recent years diverse observations about 'specific' and 'unspecific' effects of acupoints have also been experienced by a few clinical trials in the West. For instance, a randomized controlled trial illustrated from their study that acupuncture was no more effective than sham acupuncture or minimal acupuncture in reducing migraine headache and tension-types of headache, despite its effectiveness compared with no treatment [7,8]. Their results suspected that acupuncture effect may be due to unspecific physiology effects of needling, to a powerful placebo effect, or to a combination of both [7]. We argue, however, through our early investigation and the undergoing multi-center randomized controlled large-scale trial, that acupuncture does have effect in treating migraine. Through the reported studies in this article, we examine the efficacy of acupuncture for treating migraine to large samples of patients. Furthermore, we investigate whether the different efficacy of three acupoints treatment groups is due to the acupoints of different meridians or different acupoints of one meridian. We believe this trial could demonstrate that the efficacy of using acupuncture to treat migraine is *not *due to physiological effects of acupoints suspected in [7], but the real 'specific effects' of acupoints based on meridians and a collection of meridian Qi.

Central randomization is used in our trial, which is a strict, complete randomization, and ensures adequate concealment. During the whole trial period, the patients are not aware of which group they belong to, but they do know they will receive acupuncture needling treatments by either puncturing different acupoints or puncturing non-acupoints.

Before the trial started, we spent plenty of time to define the non-acupoints control groups because the location of non-acupoints were used differently compared to the previous literature in China and in the West. In most Chinese literature, the location of the non-acupoints used was selected beside the therapeutic acupoints and half between the two lines or two acupoints [15,16], while a few other reports in Western literature recorded the location of non-acupoints differently [12,17]. In our previous trials, two control groups from a consensus of acupuncture experts have been studied. So, the non-acupoints control group was defined finally according to the first part of our ***973 project***.

In a word, the purpose of this trial is to provide convinced evidence that specific effect of acupoints for treating migraine does not only exist but also differs according to the meridian and the collection of meridian Qi used.

## Competing interests

The authors declare that they have no competing interests.

## Authors' contributions

LY, LFY, YSG, LXG, TY participated in the conception and design of the trial, in plans for the analysis of the data, and in drafting the manuscript. YXG, TXP, YJ, SGJ, CXR participated in the conception and design of the trial and in drafting the manuscript. ZH, ZHX, MTT participated in the design of the trial, in plans for the analysis of the data, and in drafting the manuscript. All authors read and approved the final manuscript.

## References

[B1] Stewart WF, Shechter A, Rasmussen BK (1994). Migraine prevalence: a review of population-based studies. Neurology.

[B2] Lipton RB, Stewart WF, Diamond S, Diamond ML, Reed M (2001). Prevalence and burden of migraine in the United States: data from the American Migraine Study II. Headache.

[B3] Vickers AJ, Rees RW, Zollman CE, McCarney R, Smith C, Ellis N, Fisher P, Haselen RV (2004). Acupuncture for migraine and chronic tension headache in primary care: a large, pragmatic, randomised trial. BMJ.

[B4] Allais G, De Lorenzo C, Quirico PE, Airola G, Tolardo G, Mana O, Benedetto C (2002). Acupuncture in the prophylactic treatment of migraine without aura: a comparison with flunarizine. Headache.

[B5] Melchart D, Linde K, Fischer P, Berman B, White A, Vickers A, Allais G (2001). Acupuncture for idiopathic headache. Cochrane Database Syst Rev.

[B6] Melchart D, Thormaehlen J, Hager S, Liao J, Linde K, Weidenhammer W (2003). Acupuncture versus placebo versus sumatriptan for early treatment of migraine attacks: a randomized controlled trial. J Intern Med.

[B7] Linde K, Streng A, Jurgens S, Hoppe A, Brinkhaus B, Witt C, Wagenpfeil S, Pfaffenrath V, Hammes MG, Weidenhammer W, Willich SN, Melchart D (2005). Acupuncture for Patients With Migraine A Randomized Controlled Trial. JAMA.

[B8] Melchart D, Streng A, Hoppe A, Brinkhaus B, Witt C, Wagenpfeil S, Pfaffenrath V, Hammes M, Hummelsberger J, Irnich D, Weidenhammer W, Willich SN, Linde K (2005). Acupuncture in patients with tension-type headache: randomized controlled trial. BMJ.

[B9] International Headache Society Clinical Trails subcommittee (2000). Guidelines for controlled trials of drugs in migraine: second edition. Cephalalgia.

[B10] Jialiang Wang (2001). Clinical Epidemiology – design, measurement and evaluation.

[B11] International Headache Society (2004). International Classification of Headache Disorders, ICHD-2. Cephalalgia.

[B12] Assefi NP, Sherman KJ, Jacobsen C, Goldberg J, Smith WR, Buchwald D (2005). A Randomized Clinical Trial of Acupuncture Compared with Sham Acupuncture in Fibromyalgia. Ann Intern Med.

[B13] Chang CH, Huang JL, Ting CT, Chang CS, Chen GH (2005). Atropine-Induced HRV Alteration is Not Amended by Electroacupuncture on Zusanli. The American Journal of Chinese Medicine.

[B14] Brinkhaus B, Hummelsberger J, Kohnen R, Seufert J, Hempen CH, Leonhardy H, Nögel R, Joos S, Hahn E, Schuppan D (2004). Acupuncture and Chinese herbal medicine in the treatment of patients with seasonal allergic rhinitis: a randomized-controlled clinical trial. Allergy.

[B15] Jian T, Guang-yi Y, Shao-ying W, Feng S (1996). The preliminary investigation to effect of the electro-acupuncture to ECG ST segment of CHD. Information on Traditional Chinese Medicine.

[B16] Hu KM, Wang CP, Xie HJ, Henning J (2006). Observation on activating effectiveness of acupuncture at acupoints and non-acupoints on different brain regions. Zhongguo Zhen Jiu.

[B17] Maioli C, Falciati L, Marangon M, Perini S, Losio A (2006). Short- and long-term modulation of upper limb motorevoked potentials induced by acupuncture. Eur J Neurosci.

